# Lack of change in the respiratory quotient during oxygen supply dependence in endotoxemic shock: a subanalysis of an experimental controlled study

**DOI:** 10.5935/2965-2774.20230041-en

**Published:** 2023

**Authors:** Arnaldo Dubin, Juan Francisco Caminos Eguillor, Gonzalo Ferrara, María Guillermina Buscetti, Héctor Saúl Canales, Bernardo Lattanzio, Luis Gatti, Facundo Javier Gutierrez, Vanina Siham Kanoore Edul

**Affiliations:** 1 Cátedras de Terapia Intensiva y Farmacología Aplicada, Facultad de Ciencias Médicas, Universidad Nacional de La Plata - La Plata, Argentina

**Keywords:** Septic shock, Anaerobiosis, Oxygen consumption, Energetic metabolism, Respiration

## Abstract

**Objective:**

To evaluate if the reductions in systemic and renal oxygen consumption are associated with the development of evidence of anaerobic metabolism.

**Methods:**

This is a subanalysis of a previously published study. In anesthetized and mechanically ventilated sheep, we measured the respiratory quotient by indirect calorimetry and its systemic, renal, and intestinal surrogates (the ratios of the venous-arterial carbon dioxide pressure and content difference to the arterial-venous oxygen content difference. The Endotoxemic Shock Group (n = 12) was measured at baseline, after 60 minutes of endotoxemic shock, and after 60 and 120 minutes of fluid and norepinephrine resuscitation, and the values were compared with those of a Control Group (n = 12) without interventions.

**Results:**

Endotoxemic shock decreased systemic and renal oxygen consumption (6.3 [5.6 - 6.6] *versus* 7.4 [6.3 - 8.5] mL/minute/kg and 3.7 [3.3 - 4.5] *versus* 5.4 [4.6 - 9.4] mL/minute/100g; p < 0.05 for both). After 120 minutes of resuscitation, systemic oxygen consumption was normalized, but renal oxygen consumption remained decreased (6.3 [5.9 - 8.2] *versus* 7.1 [6.1 - 8.6] mL/minute/100g; p = not significance and 3.8 [1.9 - 4.8] *versus* 5.7 [4.5 - 7.1]; p < 0.05). The respiratory quotient and the systemic, renal and intestinal ratios of the venous-arterial carbon dioxide pressure and content difference to the arterial-venous oxygen content difference did not change throughout the experiments.

**Conclusion:**

In this experimental model of septic shock, oxygen supply dependence was not associated with increases in the respiratory quotient or its surrogates. Putative explanations for these findings are the absence of anaerobic metabolism or the poor sensitivity of these variables in detecting this condition.

## INTRODUCTION

Shock states are characterized by the failure of the cardiovascular system to meet metabolic oxygen demands. Regardless of the different hemodynamic patterns, the distinctive and common feature of shock is the presence of tissue hypoperfusion, which results in tissue hypoxia and anaerobic metabolism. Hence, the dependence of oxygen consumption (VO_2_) on oxygen delivery (DO_2_) is considered characteristic of all types of shock.^(1)^

In patients with septic shock, VO_2_/DO_2_ dependence has been repeatedly described.^(2)^ In experimental models, oxygen supply dependence has also been found at both the systemic and organ levels-such as in the gut and kidney.^(3,4)^ However, the meaning of this phenomenon is controversial. Although the fall in VO_2_ is usually considered an expression of anaerobic metabolism leading to organ dysfunction, other explanations are possible. In septic shock, while the mitochondrial ability to generate cellular adenosine-triphosphate (ATP) is decreased, this is not associated with significant organ necrosis.^(5)^ Therefore, the reduction in VO_2_ might be an adaptive response that allows survival in the face of an overwhelming insult. The suppression of nonessential functions-such as glomerular filtration rate and consequent tubular energy demand-might thus be a mechanism to avoid death by dysoxia. From this standpoint, organ failure could be a reactive and potentially protective mechanism.^(6)^

An approach that might help to elucidate the meaning of the VO_2_/DO_2_ dependence is the analysis of the respiratory quotient (RQ). The RQ is the ratio between carbon dioxide (CO_2_) production (VCO_2_) and VO_2_. In animal models of tissue hypoxia, the beginning of anaerobic metabolism is signaled by the abrupt increase in the RQ.^(7,8)^ Although VCO_2_ and VO_2_ decrease secondary to the compromise of aerobic metabolism, there is anaerobic VCO_2_ due to the buffering of protons derived from anaerobically generated acids by bicarbonate. Therefore, the relative increase in VCO_2_ in relation to VO_2_ increases the RQ.

In an experimental model of endotoxemic shock and severe kidney injury, we previously found the presence of systemic and renal oxygen supply dependence.^(4)^ The decreases in renal VO_2_ were still present after resuscitation. Notwithstanding this, the renal oxygen ratio extraction (O_2_ER) remained stable and eventually decreased, suggesting a primary reduction in metabolic oxygen needs.

The goal of this subanalysis was to evaluate whether the reductions in systemic and renal VO_2_ are associated with the development of evidence of anaerobic metabolism. For this purpose, we examined the changes in RQ and its systemic and regional surrogates, the ratios of venous-arterial CO_2_ pressure and the content difference to the arterial-venous O_2_ content difference (P_v-a_CO_2_/C_a-v_O_2_ and C_v-a_CO_2_/C_a-v_O_2_, respectively).^(9)^ Our hypothesis was that VO_2_/DO_2_ dependence is not associated with anaerobic metabolism, as reflected by the RQ and its surrogates.

## METHODS

We used original data from a previously published study.^(4)^ The local research committee approved this study [protocol P01-05-2016]. Care of animals was in accordance with the National Research Council’s Guide for the Care and Use of Laboratory Animals.

### Anesthesia and ventilation

Twenty-four sheep (24 [22 - 27] kg, median [25^th^-75^th^ percentiles]) were anesthetized with 30mg.kg^-1^ sodium pentobarbital, intubated and mechanically ventilated with a Servo Ventilator 900C (Siemens - Elema AB, Solna, Sweden) with a tidal volume of 10mL/kg, an FiO_2_ of 0.21 and a positive end-expiratory pressure of 6cmH_2_O. The initial respiratory rate was set to keep the arterial PCO_2_ between 35 - 40mmHg. This respiratory setting was maintained during the rest of the experiment. Neuromuscular blockade was performed with pancuronium bromide (0.06mg.kg^-1^). Additional pentobarbital boluses (1mg/kg) were administered hourly and when clinical signs of inadequate depth of anesthesia were evident. Analgesia was provided by fentanyl as a bolus of 2µg/kg, followed by 1µg/kg/h. These drugs were administered intravenously.

### Surgical preparation

A 7.5 French Swan - Ganz Standard Thermodilution Pulmonary Artery Catheter (Edwards Life Sciences, Irvine, CA, USA) was inserted in the right external jugular vein to obtain mixed venous samples. Catheters were placed in the descending aorta via the left femoral artery to measure blood pressure and obtain blood samples and in the inferior vena cava to administer fluids and drugs.

A midline laparotomy was performed, followed by a gastrostomy to drain the gastric contents and a splenectomy to avoid spleen contraction during shock. Perivascular ultrasonic flow probes were placed around the superior mesenteric artery and the left renal artery to measure intestinal blood flow (IBF) and renal blood flow (RBF). Catheters were introduced in the left renal and mesenteric veins to draw blood samples and to measure venous pressure. Catheters were also positioned in the abdomen for intraabdominal pressure measurement and into the bladder to monitor urinary output. To allow renal cortical videomicroscopy, the left kidney was gently decapsulated, and 5cm incision was made in the left flank of the abdominal wall. A 10- to 15cm segment of the ileum was mobilized, placed outside the abdomen, and opened 2cm on the antimesenteric border to allow examination of mucosal microcirculation. The exteriorized intestinal segment was covered and moistened, and the temperature was preserved by an external heating device. Finally, after complete hemostasis, the midline abdominal wall incision was closed, except for a short segment for externalization of the ileal loop.

### Measurements and derived calculations

Systemic VO_2_ and the RQ were measured by analysis of expired gases (MedGraphics CPX Ultima, Medical Graphics Corporation, St. Paul, MN) and adjusted to body weight.

Arterial, mixed venous, renal venous, and mesenteric venous PO_2_, PCO_2_, pH, Hb, and O_2_ saturation were measured with a blood gas analyzer and a co-oximeter in sheep mode (ABL 5 and OSM 3, Radiometer, Copenhagen, Denmark). Oxygen-derived variables were calculated by standard formulae.

Since the thermodilution method overestimates low cardiac output, the cardiac index (CI) was calculated as VO_2_ divided by the arterial-mixed venous O_2_ content difference (C_a-mv_O_2_). Oxygen delivery was calculated as the CI multiplied by the arterial O_2_ content (C_a_O_2_). Systemic O_2_ER was calculated as C_a-mv_O_2_ divided by C_a_O_2_.

Intestinal blood flow and RBF were measured by an ultrasonic flowmeter (One Channel Perivascular Flowmeter, Transonics Systems Inc., Ithaca, NY, USA) and normalized to the organ weight.

Intestinal and renal DO_2_ and VO_2_ were calculated as the product of the respective flow index multiplied by either the C_a_O_2_ or arterial-venous oxygen content difference. Intestinal and renal O_2_ER were calculated as the respective arteriovenous oxygen content difference divided by C_a_O_2_ (C_a-iv_O_2_ and C_a-rv_O_2_, respectively).

As surrogates of the systemic, renal, and intestinal RQ, we calculated the systemic, renal, and intestinal P_v-a_CO_2_/C_a-v_O_2_. In addition, the corresponding C_v-a_CO_2_/C_a-v_O_2_ values were calculated by means of the Douglas algorithm^(10)^ to assess the changes in the CO_2_ dissociation curve.

Arterial lactate was measured with a point-of-care analyzer (Stat Profile Critical Care Xpress, Nova Biomedical, Waltham, MA, USA).

Creatinine clearance was calculated as the urinary creatinine level multiplied by the urine output in 60 minutes divided by the plasma creatinine level.

### Experimental procedure

Basal measurements were taken after a period of no less than 30 minutes after blood pressure, heart rate, systemic VO_2_, and renal and intestinal flow became stable. Animals were then randomly assigned to the endotoxemic shock (n = 12) or control (n = 12) groups. In the endotoxemic shock group, shock was induced by intravenous injection of *Escherichia coli* lipopolysaccharide (5µg/kg followed by 2.5µg/kg/hour for 180 minutes). After 60 minutes of shock, 30mL/kg of 0.9% sodium chloride (NaCl) solution was infused, and norepinephrine was titrated to reach a mean arterial pressure (MAP) of 70mmHg. In the sham group, the same experimental preparation was carried out, and 0.9% NaCl was infused to maintain hemodynamic variables at basal values without further interventions. Measurements were performed at baseline (0 minutes), after 60 minutes of endotoxemic shock without resuscitation, and after 60 and 120 minutes of resuscitation. Blood temperature was kept constant throughout the study with a heating lamp.

At the end of the experiment, animals were killed with an additional dose of pentobarbital and a potassium chloride (KCl) bolus. A catheter was inserted in the superior mesenteric artery, and Indian ink was instilled through the catheter. Dyed intestinal segments were dissected, washed, and weighed. We also weighed the left kidney. Consequently, renal and intestinal VO_2_ and DO_2_ are expressed as indices based on organ weight.

### Data analysis

Because of the small numbers of animals, nonparametric tests were used. Data expressed as medians [25^th^ - 75^th^ percentiles] were analyzed using generalized estimating equations (GEE), followed by Mann‒Whitney and Wilcoxon tests with Bonferroni correction for betweenand within-group pairwise comparisons. The association of the RQ with the systemic P_v-a_CO_2_/C_a-v_O_2_ and C_v-a_CO_2_/C_a-v_O_2_ was assessed by means of the Spearman correlation. Agreement between the RQ and C_v-a_CO_2_/C_a-v_O_2_ was evaluated by the Bland and Altman method. A p value < 0.05 was considered statistically significant.

## RESULTS

**Figure 1:**
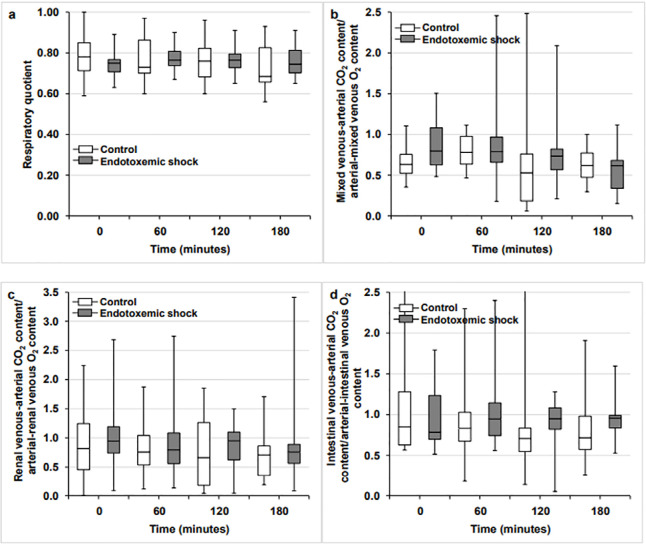
Respiratory quotient (panel A), ratio of the mixed venous-arterial carbon dioxide pressure difference to arterial-mixed venous oxygen content difference (panel B), ratio of the renal venous-arterial carbon dioxide pressure difference to arterial-renal venous oxygen content difference (panel C), and ratio of the intestinal venous-arterial carbon dioxide pressure difference to arterial-intestinal venous oxygen content difference (panel D) in the control and endotoxemic shock groups.

**Figure 2:**
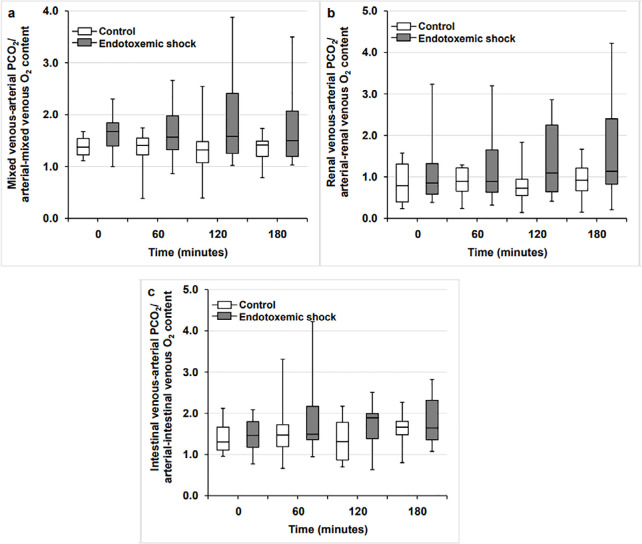
Ratio of the mixed venous-arterial carbon dioxide content difference to arterial-mixed venous oxygen content difference (panel A), ratio of the renal venous-arterial carbon dioxide content difference to arterial-renal venous oxygen content difference (panel B), and ratio of the intestinal venous-arterial carbon dioxide content difference to arterial-intestinal venous oxygen content difference (panel C) in the control and endotoxemic shock groups.

The effect of endotoxemic shock and subsequent resuscitation on systemic, regional, and microvascular hemodynamics and oxygen transport has been reported elsewhere.^(4)^ Briefly, endotoxin administration decreased blood pressure, CI, RBF, and IBF (Table 1). Systemic VO_2_ and DO_2_ fell, and O_2_ER increased. Renal VO_2_ and DO_2_ decreased, but renal O_2_ER did not change. At the intestinal level, DO_2_ was reduced but due to the increase in O_2_ER, VO_2_ remained stable (Table 1 and Figures 1S, 2S, and 3S - Supplementary Material). Microcirculatory alterations arose in the sublingual mucosa, intestinal villi and, especially, renal peritubular capillaries.^(4)^ Oliguria and severe acute kidney injury were also present (Table 1).

**Table 1:** Values of systemic, intestinal, and renal hemodynamic and oxygen transport variables in the control and endotoxemic shock groups

		Basal	60 minutes	120 minutes	180 minutes
Heart rate (beats/minute)	Control	157 [143 - 156]	160 [146 - 174]	176 [135 -190]	161 [150 - 181]
	Endotoxemic shock	155 [125 - 172]	122 [106 - 131] ^*^†	167 [156 - 200]	165 [132 - 180]
Mean arterial pressure (mmHg)	Control	80 [74 - 94]	87 [78 - 99]	93 [75 - 106]	93 [74 - 106]
	Endotoxemic shock	83 [71 - 98]	34 [31 - 40]^*^†	72 [70 - 74]†	71 [70 - 73]†
Cardiac index (mL/minute/kg)	Control	144 [123 - 168]	135 [125 - 192]	144 [122 - 174]	159 [120 - 210]
	Endotoxemic shock	138 [110 - 161]	90 [73 - 113]^*^†	174 [110 - 244]	161 [129 - 183]
Superior mesenteric artery flow (mL/minute/100g)	Control	44.0 [34.2 - 57.4]	41.3 [33.1 - 60.3]	46.0 [36.2 - 60.6]	48.8 [43.7 - 69.4]
	Endotoxemic shock	44.2 [29.1 - 67.7]	26.2 [21.9 - 47.8]^*^†	36.2 [24.9 - 52.0]	40.7 [24.5 - 63.1]
Left renal blood flow (mL/minute/100g)	Control	198 [150 - 443]	199 [157 - 394]	201 [144 - 286]	221 [170 - 221]
	Endotoxemic shock	205 [157 - 293]	131 [99 - 185]^*^†	182 [160 - 253]	174 [91 - 186]^*^†
Systemic O_2_ transport (mL/minute/kg)	Control	17.4 [16.0 - 19.1]	17.8 [15.9 - 22.3]	18.1 [16.5 - 20.5]	20.2 [15.7 - 23.4]
	Endotoxemic shock	18.2 [14.6 - 22.5]	12.3 [8.6 - 14.2]^*^†	23.3 [11.3 - 30.8]	20.0 [11.0 - 21.9]
Systemic O_2_ consumption (mL/minute/kg)	Control	7.2 [6.3 - 8.2]	7.4 [6.3 - 8.5]	7.0 [6.2 - 8.1]	7.1 [6.1 - 8.6]
	Endotoxemic shock	7.1 [6.5 - 8.1]	6.3 [5.6 - 6.6]^*^†	7.3 [5.9 - 8.1]	6.3 [5.9 - 8.2]
Systemic O_2_ extraction ratio	Control	0.40 [0.36 - 0.45]	0.40 [0.33 - 0.47]	0.41 [0.32 - 0.44]	0.39 [0.32 - 0.45]
	Endotoxemic shock	0.40 [0.29 - 0.48]	0.54 [0.46 - 0.66]^*^†	0.35 [0.27 - 0.52]	0.36 [0.30 - 0.51]
Intestinal O_2_ transport (mL/minute/100g)	Control	5.1 [4.3 - 7.6]	5.2 [4.0 - 7.6]	5.4 [4.4 - 8.3]	5.7 [4.6 - 9.5]
	Endotoxemic shock	6.0 [4.0 - 8.7]	3.4 [2.7 - 5.4]^*^	4.5 [3.1 - 7.1]	5.0 [3.2 - 7.6]
Intestinal O_2_ consumption (mL/minute/100g)	Control	2.1 [1.9 - 2.4]	1.9 [1.7 - 2.1]	2.2 [1.6 - 2.5]	2.0 [1.4 - 2.6]
	Endotoxemic shock	2.2 [1.6 - 2.9]	2.2 [1.2 - 2.5]	2.4 [1.6 - 3.1]	2.7 [1.7 -3.0]
Intestinal O_2_ extraction ratio	Control	0.42 [0.32 - 0.45]	0.35 [0.30 - 0.44]	0.33 [0.29 - 0.40]	0.29 [0.25 - 0.34]
	Endotoxemic shock	0.36 [0.30 - 0.48]	0.52 [0.37 - 0.68]^*^†	0.49 [0.38 - 0.72]^*^†	0.51 [0.40 - 0.66]^*^†
Renal O_2_ transport (mL/minute/100g)	Control	24.5 [17.1 - 62.2]	25.4 [18.9 - 51.8]	22.6 [17.6 - 38.2]	25.8 [19.2 - 35.6]
	Endotoxemic shock	28.4 [19.0 - 38.2]	15.8 [13.5 - 23.2]^*^†	23.2 [17.9 - 32.1]	20.5 [10. - 22.7]^*^
Renal O_2_ consumption (mL/minute/100g)	Control	5.1 [3.4 - 9.1]	5.4 [4.6 - 9.4]	6.1 [5.1 - 9.4]	5.7 [4.5 - 7.1]
	Endotoxemic shock	5.4 [4.0 - 8.8]	3.7 [3.3 - 4.5]^*^†	4.2 [2.7 - 5.4]†	3.8 [1.9 - 4.8]^*^†
Renal O_2_ extraction ratio	Control	0.18 [0.16 - 0.23]	0.22 [0.18 - 0.26]	0.22 [0.19 - 0.26]	0.21 [0.18 - 0.26]
	Endotoxemic shock	0.21 [0.15 - 0.24]	0.26 [0.18 - 0.36]	0.16 [0.13 - 0.20]†	0.21 [0.16 - 0.32]
Urine output (mL/minute/kg)	Control	1.2 [0.7 - 2.6]	0.95 [0.6 - 2.2]	1.0 [0.6 - 2.2]	1.2 [0.7 - 3.2]
	Endotoxemic shock	1.8 [1.1 - 2.2]	0.3 [0.2 - 0.4]^*^†	0.3 [0.2 - 0.6]^*^†	0.2 [0.1 - 0.3]^*^†
Creatinine clearance (mL/minute)	Control	46 [38 - 84]	44 [39 - 54]	51 [33 - 71]	49 [29 - 61]
	Endotoxemic shock	62 [38 - 102]	11 [4 - 25]^*^†	8 [5 - 15]^*^†	6 [1 - 13]^*^†

O_2_ - oxygen. Data are shown as the median [25-75 interquartile range].

* p < 0.05 *versus* basal. † p < 0.05 *versus* control.

Resuscitation normalized the CI and systemic VO_2_ and DO_2_. Renal blood flow and renal DO_2_ and VO_2_ remained low, whereas renal O_2_ER never increased and dropped at 60 min of resuscitation. Intestinal DO_2_ improved, but O_2_ER remained high (Table 1 and Figures 1S, 2S, and 3S - Supplementary Material). Most of the renal microvascular abnormalities appearing during shock were still present in the resuscitation period. In the intestinal and sublingual mucosa, only minor alterations persisted.^(4)^

In the endotoxemic group, hyperlactatemia and increased anion gap metabolic acidosis developed during resuscitation (Table 2).

**Table 2:** Values of arterial blood gases in the control and endotoxic shock groups

		Basal	60 minutes	120 minutes	180 minutes
Hemoglobin (g/L)	Control	10.0 [9.4 - 11.1]	10.1 [9.4 - 10.7]	9.7 [9.3 - 10.5]	9.6 [9.1 - 10.3]
	Endotoxemic shock	10.7 [10.1 - 11.4]	10.7 [10.1 - 11.8]	10.2 [9.3 - 11.3]	10.5 [9.2 - 11.3]
Arterial pH	Control	7.41 [7.38 - 7.46]	7.42 [7.33 - 7.47]	7.40 [7.34 - 7.46]	7.40 [7.33 - 7.44]
	Endotoxemic shock	7.43 [7.40 - 7.45]	7.37 [7.34 - 7.42]	7.30 [7.24 - 7.34]^*^†	7.29 [7.21 - 7.35]^*^†
Arterial PCO_2_ (mmHg)	Control	37 [35 - 39]	35 [34 - 38]	35 [32 - 37]	36 [33 - 37]
	Endotoxemic shock	37 [35 - 38]	34 [33 - 36]	37 [34 - 40]	37 [34 - 42]
Arterial PO_2_ (mmHg)	Control	82 [76 - 93]	83 [70 - 90]	85 [74 - 94]	81 [70 - 91]
	Endotoxemic shock	87 [81 - 92]	79 [68 - 94]	79 [69 - 94]	69 [59 - 92]
Arterial bicarbonate (mEq/L)	Control	23 [21 - 25]	22 [20 - 25]	20 [19 - 25]	21 [18 - 22]
	Endotoxemic shock	24 [22 - 26]	20 [19 - 24	18 [17 - 21]^*^†	18 [17 - 19]^*^†
Arterial base excess (mEq/L)	Control	- 1 [- 4 - 2]	- 3 [ -5 - 1]	- 5 [-5 - 1]	- 5 [-6 - -1]
	Endotoxemic shock	1 [-2 - 2]	-4 [- 6 - 0]^*^†	-7 [-9 - -4]^*^†	-8 [-9 - -4]^*^†
Arterial anion gap (mEq/L)	Control	16 [15 - 18]	15 [15 - 16]	15 [13 - 17]	14 [14 - 15]
	Endotoxemic shock	17 [14 - 17]	17 [14 - 20]	20 [16 - 22]^*^†	20 [15 - 24]^*^†
Arterial lactate (mmol/L)	Control	2.3 [1.8 - 3.3]	2.5 [1.8 - 3.1]	2.1 [1.3 - 3.2]	1.7 [1.1 - 3.2]
	Endotoxemic shock	2.3 [2.0 - 3.3]	3.6 [2.9 - 4.3]	4.7 [2.7 - 5.4]^*^†	5.0 [2.9 - 6.6]^*^†

PCO_2_ - partial pressure of carbon dioxide; PO_2_ - partial pressure of oxygen. Data are shown as the median [0.25 - 0.75 interquartile range].

* p < 0.05 *versus* basal. † p < 0.05 *versus* control.

In both groups, RQ did not change throughout the experiments. The systemic, renal, and intestinal P_v-a_CO_2_/C_a-v_O_2_ and C_v-a_CO_2_/C_a-v_O_2_ also remained unchanged (Figures 1 and 2).

CO_2_ - carbon dioxide; O_2_ - oxygen.

PCO_2_ - partial pressure of carbon dioxide; O_2_ - oxygen.

The RQ showed a weak but statistically significant correlation with P_mv-a_CO_2_/C_a-mv_O_2_ (rs = 0.23, p = 0.02). The RQ had no correlation with C_mv-a_CO_2_/C_a-mv_O_2_ (r_s_ = 0.10, p = 0.36). Bland and Altman analysis showed a bias of -0.04, a precision of 0.41, and 95% limits of agreement of 1.60 between RQ and C_mv-a_CO_2_/C_a-mv_O_2_ (Figures 4S, 5S, and 6S - Supplementary Material).

## DISCUSSION

The main finding of this study was that the RQ and its systemic and regional surrogates did not change, despite the severe hemodynamic compromise with oxygen supply dependence, tissue hypoperfusion, and acute kidney injury produced by the administration of endotoxin. The lack of increase in the RQ and its surrogates might suggest the absence of anaerobic metabolism but also the inability of these variables to reflect tissue hypoperfusion.

Our experimental model is relevant and resembles many components of human septic shock, including derangements in systemic and microvascular hemodynamics. In addition, it produced severe renal failure, which was unresponsive to resuscitation. Remarkably, the renal O_2_ER never increased and eventually dropped at 60 minutes after the start of the resuscitation period.

The concurrent alterations in RBF and peritubular microcirculation could be considered the cause of renal failure or a reflex compensation for metabolic shutdown. Since previous studies could not detect overt necrotic lesions,^(11)^ septic acute kidney injury has been related to bioenergetic failure. This hypothesis states that mitochondrial dysfunction and insufficient adenosine triphosphate lead to reduced cellular metabolism. Organ failures might thus be primarily functional rather than structural. Indeed, this could act as a potentially protective, reactive mechanism against inflammatory stress.^(6)^ In an experimental study, proximal tubular cells exposed to endotoxin developed an irreversible reduction in VO_2_ as a sign of pathologic metabolic downregulation.^(12)^ Even though this process is usually described at several hours or days after septic challenge, the intravenous administration of endotoxin is associated with almost immediate reductions in the intestinal redox state of mitochondrial cytochrome aa_3_.^(13)^ Likewise, within 1 hour of endotoxin exposure, renal cells show decreased expression of genes involved in mitochondrial processes.^(14)^ The lack of changes in the RQ and systemic and regional P_v-a_CO_2_/C_a-v_O_2_ and C_v-a_CO_2_/C_a-v_O_2_ might be linked to bioenergetic failure, but this is merely speculative because mitochondrial function was not assessed in our study.

Normal RQ values range from 0.67 to 1.10, which depends on the type of substrate utilized.^(15)^ For this reason, a sharp increase-rather than isolated high values-of the RQ signals the beginning of anaerobic metabolism during progressive exercise load and during reductions in oxygen transport in critically ill patients.^(16)^ Acute increases in the RQ have been described in ischemic, hypoxic, and anemic hypoxia.^(7,8,17,18)^

Another explanation for the lack of changes in the RQ during endotoxemic shock might be a switch in the source of energy, *i.e.*, from carbohydrates to lipids. In this case, however, the RQ should be lower during resuscitation.

In septic shock, there are conflicting results about the behavior of the RQ and its surrogates. In our study, these variables remained constant. In a similar model of endotoxemic shock with systemic and intestinal oxygen supply dependence, we found that the corresponding C_v-a_CO_2_/C_a-v_O_2_ did not increase.^(19)^ Apart from ours, only two studies, which were carried out in rodent models of sepsis, have assessed the RQ calculated from the measurement of expired gases during VO_2_/DO_2_ dependence.^(20,21)^ In contrast to our results, endotoxin injection resulted in an increase in the RQ. This discrepancy could be related to the species studied (rats, guinea pigs, and sheep). Severely hypodynamic murine models of sepsis have been considered poorly representative of human sepsis^(5)^ Another explanation might reside in the fact that our animals were on mechanical ventilation, whereas the rodents breathed spontaneously. Spontaneous breathing is a major contributor to the development of muscle anaerobic metabolism and lactic acidosis in shock states, regardless of hemodynamic changes.^(22,23)^

In another study performed in septic pigs, the systemic and regional P_v-a_CO_2_/C_a-v_O_2_ and C_v-a_CO_2_/C_a-v_O_2_ did not change, but VO_2_/DO_2_ dependence was absent.^(24)^ In patients with septic shock, the RQ had a similar time course in survivors and nonsurvivors.^(25,26)^ Although mortality was associated with temporal decreases in VCO_2_ and VO_2_, the RQ was stable over time. In contrast, in perioperative shock, the RQ was a predictor of hyperlactatemia and complications.^(27-29)^

P_v-a_CO_2_/C_a-v_O_2_ and C_v-a_CO_2_/C_a-v_O_2_ have been used as surrogates for the RQ. Experimental studies have shown that both variables increase during states of ischemic, hypoxic, and anemic hypoxia.^(30,31)^ In patients with septic shock, a P_v-a_CO_2_/C_a-v_O_2_ higher than 1.4 was a predictor of mortality, hyperlactatemia, and oxygen supply dependence.^(9)^ Nevertheless, P_v-a_CO_2_/C_a-v_O_2_ can increase before the start of VO_2_/DO_2_ dependence or persist at an elevated level after correction of tissue hypoxia.^(30-32)^ Factors that enhance the dissociation of CO_2_ from hemoglobin, such as anemia, metabolic acidosis, and the Haldane effect, can account for the increase in P_v-a_CO_2_/C_a-v_O_2_. In our study, P_v-a_CO_2_/C_a-v_O_2_ only showed a weak correlation with the RQ. The calculation of C_v-a_CO_2_/C_a-v_O_2_ should overcome these difficulties, but this approach could also be misleading. In the validation of the Douglas algorithm, an excellent correlation between the tonometric and calculated CO_2_ content was found.^(10)^ However, using data from the abovementioned study, the 95% limits of agreement between the measured and calculated CO_2_ contents are as large as 4.7mL/100mL. In addition, there is a propagation error linked to the calculation of C_v-a_CO_2_. For these reasons, this calculation can occasionally result in spurious negative values of C_v-a_CO_2_. Accordingly, we found no correlation and wide 95% limits of agreement between the RQ and C_v-a_CO_2_/C_a-v_O_2_. Previous experimental and clinical studies showed that P_v-a_CO_2_/C_a-v_O_2_ is a misleading surrogate for the RQ.^(28,31,32)^ It might exhibit high sensitivity but low specificity to detect increases in the RQ. Despite the high sensitivity, this variable remained markedly stable in our experiments, even in the presence of systemic and renal oxygen supply dependence.

The increase in the RQ in anaerobic states results from the anaerobic VCO_2_ produced by bicarbonate buffering of anaerobically generated acids, such as lactate.^(16)^ In our experiments, lactate only showed marginal increases in the initial phase of shock, which is congruent with the lack of changes in the RQ and its surrogates and might imply the preservation of aerobic metabolism. In contrast, after the restoration of systemic VO_2_ and DO_2_ by fluid and norepinephrine resuscitation, severe hyperlactatemia and metabolic acidosis secondary to an increased anion gap arose. A large body of evidence shows that hyperlactatemia in septic shock, especially after the normalization of blood pressure and cardiac output, depends mainly on the increased aerobic glycolysis secondary to catecholamine stimulation of Na^+^/K^+^-ATPase activity.^(33)^ Furthermore, energetic reprogramming from fatty acid oxidation and oxidative phosphorylation toward aerobic glycolysis might be contributing factors.^(34)^

This study has weaknesses. Secondary analyses pose inherent limitations that have been subject to critiques.^(35)^ Additionally, endotoxemic shock might not completely resemble human septic shock. In addition, our research lacks measurements of tissue oxygenation, bioenergetics, and mitochondrial function. Therefore, we could not completely rule out the occurrence of anaerobic metabolism. Another drawback is the lack of histologic examinations.

## CONCLUSION

In this sheep model of septic shock, systemic, regional, and microcirculatory hypoperfusion; the dependence of systemic and renal oxygen consumption on oxygen delivery; and acute kidney injury were not associated with increases in the respiratory quotient or its systemic and regional surrogates. These findings might suggest the absence of anaerobic metabolism or a poor ability of these variables to detect such conditions. In any case, this monitoring failed to reflect the abnormalities in tissue perfusion and organ function. Consequently, our results might challenge the usefulness of this monitoring in patients with septic shock. Further studies should explore the relationship between these findings and the presence of bioenergetic failure.
